# Painful Disorders of Gut‐Brain Interaction Are More Associated With Worse Health‐Related Quality of Life and Psychological Disorders Than Non‐Painful Disorders in Latin American Countries

**DOI:** 10.1111/nmo.70194

**Published:** 2025-11-10

**Authors:** Rômulo Marx, Antonio Barros Lopes, Rafael da Veiga Chaves Picon, Suzi Alves Camey, Olafur Palsson, Shrikant I. Bangdiwala, Ami D. Sperber, Albis Hani, Luis Bustos Fernandez, Max Schmulson, Carlos Francisconi

**Affiliations:** ^1^ Gastroenterology Division, Hospital de Clínicas de Porto Alegre Universidade Federal Do Rio Grande Do Sul Porto Alegre Brazil; ^2^ Graduate Program: Sciences in Gastroenterology and Hepatology, School of Medicine Universidade Federal do Rio Grande do Sul (UFRGS) Porto Alegre Brazil; ^3^ Hospital de Clínicas de Porto Alegre Universidade Federal do Rio Grande do Sul Porto Alegre Brazil; ^4^ Division of Gastroenterology and Hepatology University of North Carolina North Carolina USA; ^5^ Department of Health Research Methods, Evidence and Impact McMaster University Hamilton Ontario Canada; ^6^ Faculty of Health Sciences Ben‐Gurion University of the Negev Beer‐Sheva Israel; ^7^ Gastroenterology Unit, Hospital Universitario San Ignacio Pontificia Universidad Javeriana Bogotá Colombia; ^8^ Centro Médico Bustos Fernández Buenos Aires Argentina; ^9^ Laboratory of Liver, Pancreas and Motility (HIPAM)‐unit of Research in Experimental Medicine, Faculty of Medicine Universidad Nacional Autónoma de México (UNAM) Mexico City Mexico

**Keywords:** DGBI, functional disorders, HRQoL, psychosocial impact, Rome Foundation Global Epidemiology Study

## Abstract

**Background:**

Disorders of gut‐brain interaction (DGBI) are associated with reduced health‐related quality of life (HRQoL) and psychological disorders. Among individuals with DGBI, abdominal pain correlates with increased healthcare‐seeking and analgesic use. This study aimed to evaluate the influence of pain as a cardinal symptom on HRQoL and psychological disorders.

**Methods:**

This is a sub‐analysis of data from four Latin American countries included in the Rome Foundation Global Epidemiology Study (RFGES). DGBI were classified into (1) painful DGBI, including individuals with diagnoses characterized by pain as a primary symptom, and (2) non‐painful DGBI, including individuals with only non‐painful diagnoses. Prevalence rates, healthcare‐seeking behavior, HRQoL (Patient‐Reported Outcomes Measurement Information System Global‐10 [PROMIS Global‐10]), anxiety and depression (Patient Health Questionnaire‐4 [PHQ‐4]) and somatization (Patient Health Questionnaire‐12 [PHQ‐12]) were compared.

**Key Results:**

A total of 8069 participants from the four countries completed the RFGES online survey, including 1132 in the painful group and 1720 in the non‐painful group. Participants with painful DGBI more commonly sought healthcare at least monthly compared to those with non‐painful disorders (18.6% vs. 14.9%). Painful disorders were associated with significantly lower HRQoL scores and higher PHQ‐4 and PHQ‐12 scores, both in unadjusted and adjusted analyses (sex, age, education, and community size).

**Conclusion and Inferences:**

In four Latin American countries, individuals with painful DGBI were more likely to seek healthcare, had worse HRQoL and exhibited greater psychological distress compared to those with non‐painful DGBI. These findings highlight the need for targeted interventions for individuals with painful DGBI symptoms.

## Graphical abstract

To investigate the relevance of the clinical variable pain in healthcare‐seeking behavior among individuals with painful and non‐painful DGBI and to evaluate its impact on health‐related quality of life (HRQoL) and psychological disorders in the four Latin American countries (Argentina, Brazil, Colombia, Mexico) that were included in the RFGES. Individuals with painful DGBI seek medical care more frequently and exhibit worse quality of life and higher psychological symptom scores compared to those with non‐painful disorders.

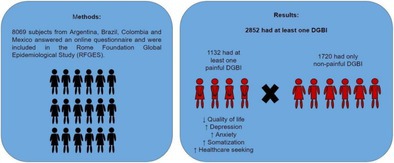

**Summary**

- Disorders of gut‐brain interaction (DGBI) are common in the Four Latin american countries and non‐painful disorders represent most of it .
- Individuals with painful DGBI seek healthcare more frequently on a monthly basis than individuals with exclusively non‐painful disorders.
- The impact on health‐related quality of life and psychological symptoms when measured by validated scores is greater in individuals with painful DBGI.
- Individuals with painful dgbi may require special attention in order improve long‐term well‐being.

## 1 Introduction

Disorders of gut‐brain interaction (DGBI) affect approximately 40% of the global population and are associated with increased direct and indirect costs, as well as frequent healthcare‐seeking [1, 2]. A positive diagnosis is made using a set of symptoms established by the Rome IV criteria [3]. The pathophysiology of DGBI is known to be diverse: alterations in the intestinal microbiota and mucosa, dysmotility [4], visceral hypersensitivity [5] and aberrant pain processing and signaling [6, 7] may be involved.

Quality of life and psychological factors have been systematically studied in patients with DGBI. The Rome Foundation Global Epidemiology Study (RFGES) [1] demonstrated that individuals with any form of DGBI exhibited lower quality of life scores compared to those without such diagnoses. Moreover, individuals with overlapping DGBI reported progressively worse scores as the number of affected anatomical regions increased [8]. Among the most common psychiatric comorbidities in these individuals are anxiety and depressive disorders [9]. Patients with both DGBI and psychiatric comorbidities experience an even greater impact on their physical and mental quality of life [10], in addition to impairments in productivity, work performance and daily activities, particularly among those with multiple affected anatomical regions and disorders characterized by pain as a symptom [11].

Abdominal pain is a major cause of seeking outpatient and emergency medical care among patients with DGBI, and its presence correlates positively with the use of medications targeting gastrointestinal and psychological disorders [12, 13, 14, 15]. However, data on this regard are absent in Latin America. Therefore, the objective of this study was to investigate the relevance of the clinical variable pain in healthcare‐seeking behavior among individuals with painful and non‐painful DGBI, and to evaluate its impact on health‐related quality of life (HRQoL) and psychological disorders in the four Latin American countries (Argentina, Brazil, Colombia, Mexico) that were included in the RFGES.

## 2 Materials and Methods

This is a sub‐analysis of the data obtained from the four Latin American countries that were included in the RFGES. Recruitment and study methodology have been described in detail in earlier publications [16]. In summary, the RFGES included data collection on: (1) gastrointestinal symptoms using the Rome IV diagnostic questionnaire; (2) health‐related quality of life with the Patient‐Reported Outcomes Measurement Information System Global‐10 (PROMIS Global‐10) [17]; (3) medication use; (4) physician visits; (5) anxiety/depression scores using the Patient Health Questionnaire‐4 (PHQ‐4) [18] and (6) somatization using the Patient Health Questionnaire‐12 (PHQ‐12) in 33 countries across six continents [19]. The PHQ‐12 questionnaire was composed of the PHQ‐15 questionnaire without three questions related to gastrointestinal symptoms. The participants included in the present analysis completed the online survey (RFGES).

A minimum sample size of 2000 respondents per country was established, with pre‐defined demographic quotas of 50% women and 50% men, distributed by age as follows: 40% ages 18–39 years, 40% ages 40–64 years, and 20% ages 65 years and older, from Argentina, Brazil, Colombia, and Mexico, and the questionnaire responses were collected between April 2017 and June 2018, with varying intervals across countries. Data collection was conducted by the University of North Carolina at Chapel Hill in the United States, with research participants recruited for the study by Qualtrics Inc. (Utah, USA). The original research was submitted to ethics committees in all participating countries and approval was granted for the use of the data in future projects, including this study.

For this sub‐analysis, the 22 possible diagnoses according to the DGBI definitions [1] were divided into two groups: painful DGBI and non‐painful DGBI, based on the presence or absence of pain among the defining diagnostic symptoms. Individuals with overlapping painful and non‐painful DGBI were included in the painful DGBI group. The classification of disorders into painful and non‐painful DGBI is provided in Table S1. National prevalence rates for painful and non‐painful DGBI (percentages, with a 95% Confidence Interval [CI]) were calculated for Argentina, Brazil, Colombia, and Mexico.

### 2.1 Statistical Analysis

Chi‐square tests were performed to compare the frequency of healthcare‐seeking behavior between individuals in the painful and non‐painful DGBI groups. For each comparison group (painful and non‐painful DGBI), mean scores for the PHQ‐4, PHQ‐12 and PROMIS Global‐10 physical and mental components were estimated and adjusted for age, sex, number of years of education and community size (priori‐defined covariates) using multiple linear regression. Mean HRQoL and psychometric scores were also compared in individuals with only non‐painful DGBI, only painful DGBI and overlapping painful and non‐painful DGBI. All group comparisons were two‐tailed with a critical alpha of 0.05.

## 3 Results

### 3.1 Study Population

Among the 8069 individuals interviewed across the four Latin American countries, 2852 (35.3%) met the diagnostic criteria for at least one DGBI. The overall mean age was 42.5 ± 16.3 years and the study populations had a predominance of women (60.5%). Most participants resided in cities with populations exceeding 50,000 (83.8%). The overall prevalence of non‐painful disorders was higher (21.3%) compared to painful disorders (14.0%). The prevalences of painful and non‐painful DGBI in the four countries included are summarized in Table 1.

**TABLE 1:** Nationwide pooled prevalence rates (% and 95% CI) of painful and non‐painful dgbi in patients across four Latin American countries (Argentina, Brazil, Colombia, and Mexico) (*N* = 8069).

Country		Painful DGBI		Non‐painful DGBI	
	*N*	*n*	Prevalence (%, 95% CI)	*N*	Prevalence (%; 95% CI)
Overall	8069	1132	14.03 (13.28–14.81)	1720	21.32 (20.43–22.23)
Argentina	2057	278	13.51 (12.07–15.07)	462	22.46 (20.67–24.33)
Brazil	2004	312	15.57 (14.01–17.23)	436	21.76 (19.97–3.63)
Colombia	2007	266	13.25 (11.80–14.82)	417	20.78 (19.02–22.62)
Mexico	2001	276	13.79 (12.31–15.38)	405	20.24 (18.50–22.07)

### 3.2 Health Care Seeking Among Patients With Painful and Non‐Painful DGBI

Seeking medical care was frequent across both groups, with 78.6% of individuals reporting seeking medical care more than once a year (Table 2). Individuals with painful DGBI sought medical care more frequently (“at least once a month”) than those with non‐painful DGBI: 18.6% for painful DGBI vs. 14.9% for non‐painful DGBI (Table 2; Table S2), representing a 24.8% relative increase in the frequency of consultations “at least once a month” in individuals with painful DGBI.

**TABLE 2:** Descriptive characteristics of sex, age, years of education, and community size by painful and non‐painful DGBI in four Latin American countries (Argentina, Brazil, Colombia, and Mexico; *n* = 2852).

	Painful DGBI (*N* = 1132)	Non‐Painful DGBI (*N* = 1720)	Overall (*N* = 2852)
Sex—*n* (%)			
Male	415 (36.7)	711 (41.3)	1126 (39.5)
Female	717 (63.3)	1009 (58.7)	1726 (60.5)
Age (in years)			
Mean (SD)	41.4 (15.0)	43.2 (17.1)	42.5 (16.3)
Number of years of education completed			
Mean (SD)	14.2 (4.67)	14.7 (4.53)	14.5 (4.59)
Missing—*n* (%)	57 (5.0)	77 (4.5)	134 (4.7)
Community size—*n* (%)			
More than 50,000 inhabitants	933 (82.4)	1456 (84.7)	2389 (83.8)
Less than or equal to 50,000 inhabitants	199 (17.6)	264 (15.3)	463 (16.2)
How often do you go to a doctor for your health?—*n* (%)			
At least once a month	210 (18.6)	257 (14.9)	467 (16.4)
A few times a year	571 (50.4)	917 (53.3)	1488 (52.2)
Once a year	184 (16.3)	266 (15.5)	450 (15.8)
Less than once a year	142 (12.5)	246 (14.3)	388 (13.6)
Never	25 (2.2)	34 (2.0)	59 (2.1)

### 3.3 Health‐Related Quality of Life and Psychological Disorders

In the unadjusted analysis, the mean PROMIS Global‐10 physical and mental health scores were numerically lower in the painful DGBI group, indicating poorer quality of life among subjects with painful DGBI. Conversely, mean PHQ‐12 and PHQ‐4 scores were higher, suggesting greater somatization and anxiety/depression, respectively, in the same group. These differences in mean scores persisted even after adjusting for sex, age, years of education, and community size (Table 3).

**TABLE 3:** Crude and adjusted means of PROMIS Global‐10 scores and PHQ‐4 for patients with painful and non‐painful DGBI in four Latin American countries (Argentina, Brazil, Colombia, and Mexico; *n* = 2852).

	Analysis	Painful DGBI (*n* = 1132)	Non‐painful DGBI (*n* = 1720)	*p* ^e^
		Mean (95% CI)	Mean (95% CI)	
PROMIS Global‐10 Mental^a^	Crude	12.61 (12.43–12.79)	13.64 (13.50–13.78)	< 0.001
	Adjusted	12.87 (12.19–13.56)	13.81 (13.14–14.48)	0.064
PROMIS Global‐10 Physical^b^	Crude	12.88 (12.73–13.04)	13.90 (13.78–14.02)	< 0.001
	Adjusted	12.94 (12.37–13.51)	13.90 (13.33–14.46)	0.025
PHQ12^c^	Crude	8.16 (7.93–8.39)	6.39 (6.23–6.55)	< 0.001
	Adjusted	7.76 (6.97–8.55)	6.16 (5.38–6.93)	0.007
PHQ4^d^	Crude	4.74 (4.55–4.93)	3.44 (3.31–3.57)	< 0.001
	Adjusted	4.33 (3.66–4.99)	3.15 (2.49–3.81)	0.018

*Note:* Adjusted by sex + age + years of education + community size greater than 50,000 inhabitants.

^a^ Global Mental Health component score—PROMIS Global‐10.

^b^ Global Physical Health component score—PROMIS Global‐10.

^c^ PHQ‐12 Somatic Symptom Scale score (excluding questions about gastrointestinal tract).

^d^ PHQ‐4 score (anxiety and depression questionnaire).

^e^ Student's *t*‐test *p*‐values for comparison of means.

**FIGURE 1:**
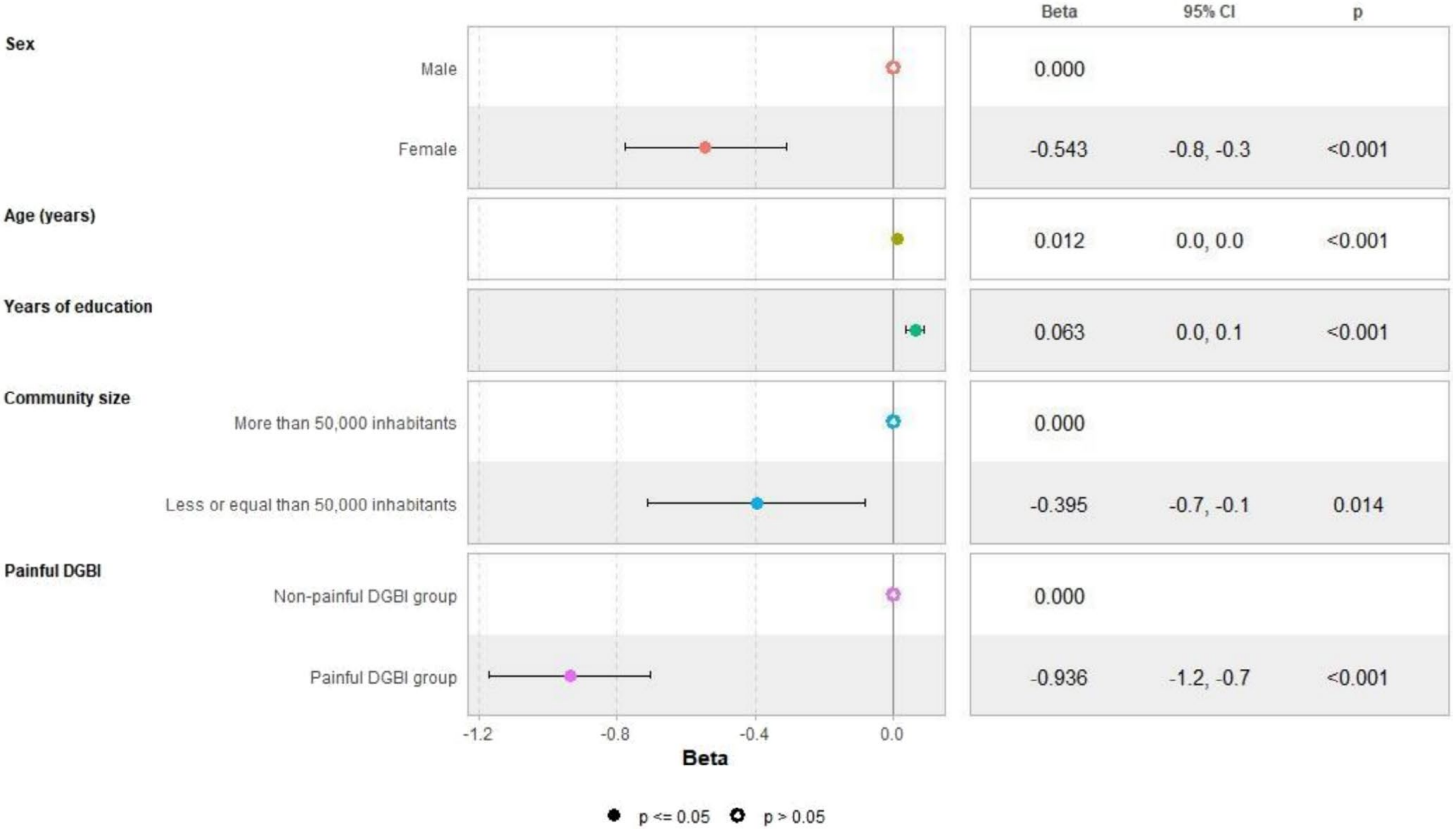
Painful DGBI and sociodemographic factors associated with PROMIS‐10 Global Mental Health component score.

**FIGURE 2:**
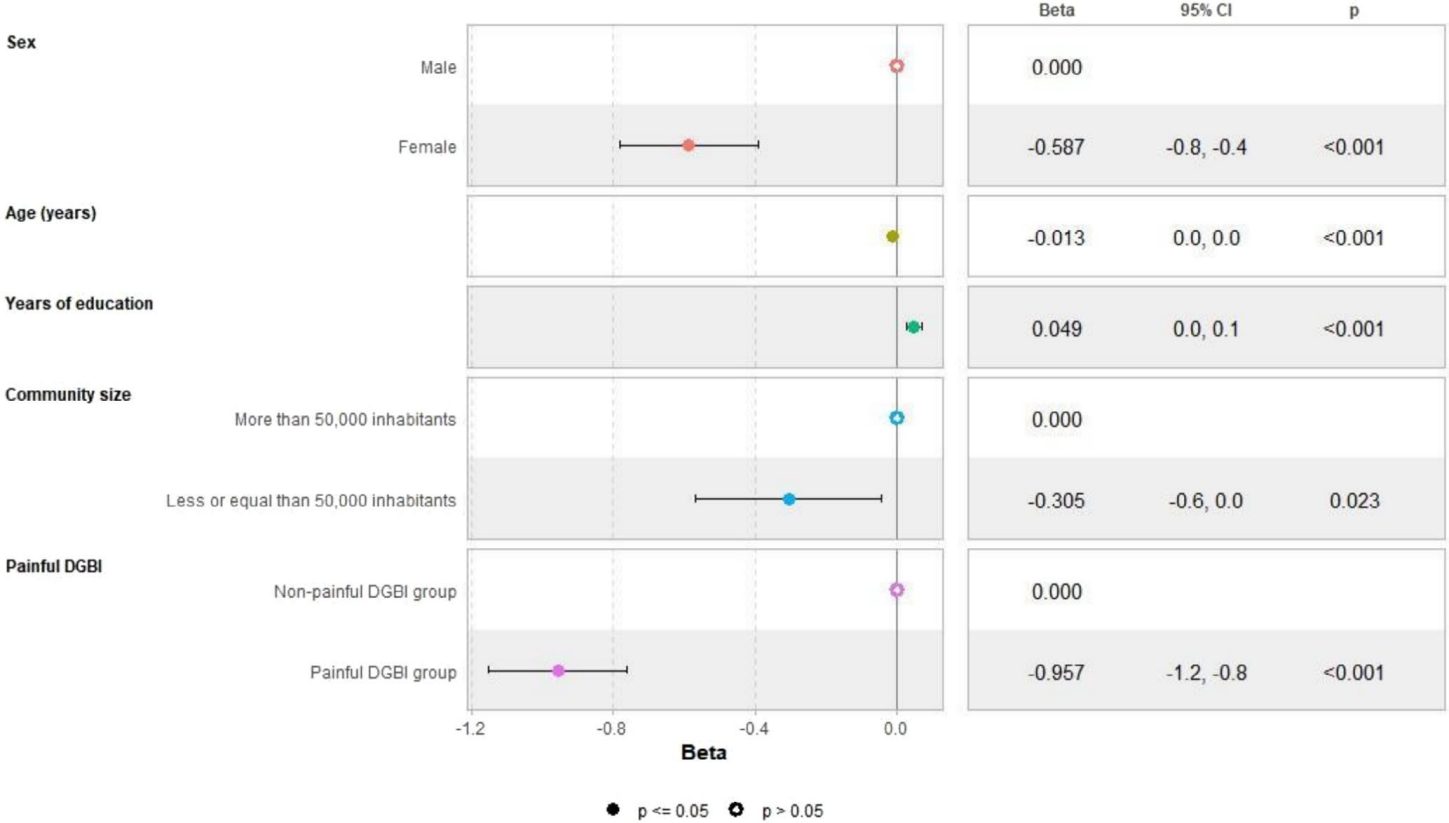
Painful DGBI and sociodemographic factors associated with PROMIS Global‐10 Physical Health component score.

**FIGURE 3:**
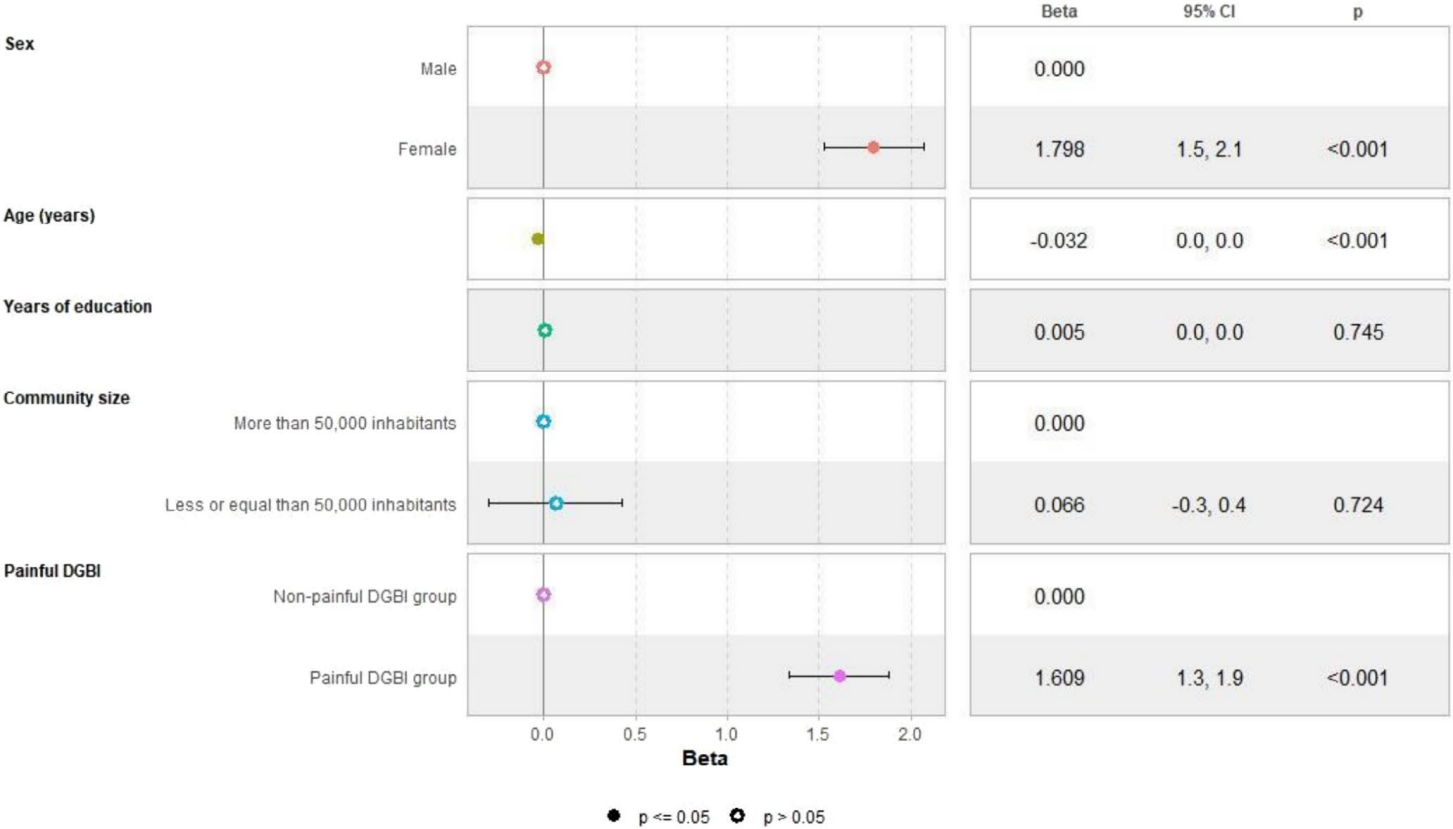
Painful DGBI and sociodemographic factors associated with anxiety and depression questionnaire PHQ‐4 score.

**FIGURE 4:**
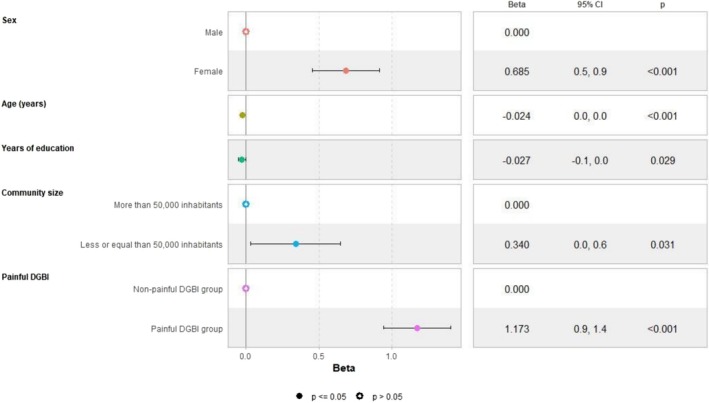
Painful DGBI and sociodemographic factors associated with PHQ‐12 Somatic Symptom Scale score.

In the adjusted linear regression models, female sex and community size with fewer than 50,000 inhabitants negatively impacted HRQoL. When painful disorders were included as a factor in the models, their presence negatively influenced all studied outcomes, with a more significant impact than either female sex or community size on both mental (Figure 1) and physical HRQoL (Figure 2). These factors were also associated with numerically higher anxiety/depression scores (Figure 3), representing higher psychological distress. Female sex emerged as an independent predictor for somatization, with a slightly greater effect than the presence of painful DGBI (Figure 4).

### 3.4 Comparison Between Painful DGBI as the Sole Manifestation or Overlapping With Non‐Painful Symptoms

**FIGURE 5:**
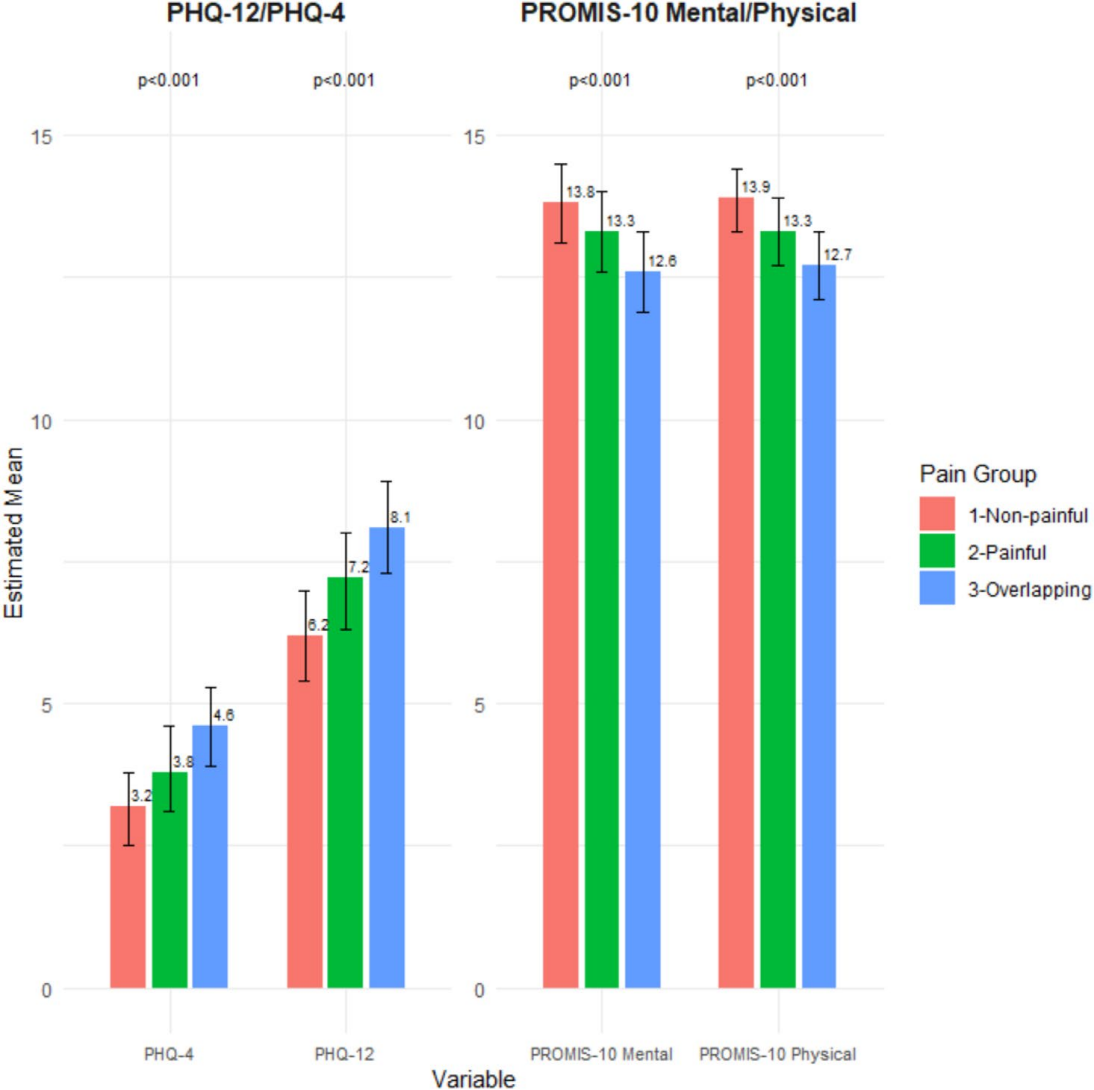
Comparison of psychological distress (PHQ‐4 and PHQ‐12) and HRQoL (PROMIS‐10 Mental and PROMIS‐10 Physical) between individuals with non‐painful disorders only, only painful disorders and overlapping painful and non‐painful disorders (Student's *t*‐test *p*‐values for comparisons of adjusted means: painful vs. non‐painful and both vs. non‐painful).

The presence of painful DGBI overlapping with non‐painful DGBI was consistently associated with worse physical and mental health quality of life scores, both in unadjusted and adjusted analyses. This same impact was also observed in psychometric scores, where the overlap was linked to higher PHQ‐4 and PHQ‐12 scores. Compared to subjects only fulfilling criteria for non‐painful DGBI, those with only painful DGBI had a higher burden of psychological symptoms and poorer quality of life (Figure 5), although to a lesser extent than in those with the overlap of both types of disorders.

## 4 Discussion

Our findings demonstrate that the presence of gastrointestinal pain among the defining symptoms of DGBI is associated with increased frequency of medical visits, poorer quality of life and higher anxiety and depression scores across four Latin American countries. To our knowledge, this is the first study to evaluate healthcare‐seeking behavior in individuals fulfilling DGBI criteria by dividing them into two groups: painful and non‐painful DGBI.

In the sample studied, individuals with painful DGBI had almost a 25% relative increase in doctor visits (at least monthly) compared to those with non‐painful DGBI. A similar finding was reported by Talley et al. [20] in a population‐based study in Australia on individuals with irritable bowel syndrome (IBS), which identified severity and duration of abdominal pain as predictors of healthcare‐seeking behavior. The profile of healthcare service utilization has also been studied in patients with functional dyspepsia, where those seeking medical care reported more severe or frequent abdominal pain compared to those who did not [21]. Similarly, a Chinese study involving individuals with IBS in a tertiary hospital associated abdominal pain or discomfort with healthcare‐seeking behavior throughout the disease course, as well as dissatisfaction with the medical care received [22].

DGBI are classified traditionally according to the Rome criteria, based on the anatomical region of the digestive tract affected, and treatments are symptom‐focused for each diagnosis. However, DGBI have a heterogeneous pathophysiology and exhibit high rates of overlap, with worse quality of life scores and increased symptom severity as individuals accumulate multiple disorders within the gut‐brain axis [8, 23]. Burton‐Murray et al. [14] studied the interactions between DGBI symptoms using network analysis and identified strong interactions between abdominal pain and thoracic, epigastric, and rectal pain. Additionally, a community‐based analysis revealed that painful symptoms formed the largest group, with interactions involving all anatomical regions of DGBI. Pain is a debilitating symptom that significantly impacts physical functioning, mood, productivity, and sleep [24]. Chronic abdominal (particularly in females), musculoskeletal, and pelvic pain are associated with higher scores of depression and anxiety [25, 26, 27]. In our study, evaluating subjects from the general population from Latin American countries with or without painful disorders, we observed that those in the painful DGBI group exhibited worse HRQoL scores and higher levels of psychological distress (anxiety, depression, and somatization). Similarly, Frändemark et al. [11], using data from the RFGES across eight countries, also classified DGBI into painful and non‐painful categories and found that painful disorders are associated with greater impairment in work productivity and daily activities.

In our study, female sex was an independent factor associated with worse quality of life scores and more psychological symptoms. A systematic review demonstrated that female sex is a risk factor for the development of painful DGBI (odds ratio: 1.56; 95% CI 1.4–1.7) [28]. In patients with IBS, female sex was linked to poorer specific quality of life scores and greater severity across most symptoms assessed, although abdominal pain affected both sexes equally [29]. These findings are consistent with data from the RFGES, which showed that women with and without DGBI had worse physical and mental quality of life scores [10]. Women also exhibited higher rates of DGBI overlap (16.7% vs. 11.2%), correlating positively with healthcare use and psychological disorders (anxiety, depression, and somatization) [30]. This effect may, in part, be explained by the influence of hormones such as estrogen on the digestive system, leading to reduced motility and modulation of sensitivity via both peripheral and central mechanisms [31].

In our study, smaller community size was associated with greater anxiety and depression scores and lower quality of life. This topic has been addressed in previous studies with mixed findings. A North American study found that individuals living in urban areas (> 75,000 inhabitants) were 25% more likely to receive prescriptions for anxiolytic medications compared to those living in rural areas (< 2250 inhabitants) [32]. Another study, using four independent datasets from the United States, observed that larger cities exhibited lower per capita rates of depression, possibly due to denser social support networks that facilitate access to resources promoting well‐being and healthcare [33]. Interestingly, individuals with DGBI living in smaller communities (< 2500 inhabitants) showed a 16% (95% CI 5%–28%) higher prevalence of prescription pain medications [15]. The differences observed in the present study compared to previous findings could be attributed to limited internet access in smaller communities within the studied countries, potentially introducing measurement bias. Another possible explanation is that smaller communities may have fewer healthcare providers prepared to manage complex conditions such as DGBI adequately.

Our results further demonstrate that the overlap between painful and non‐painful DGBI in the four countries of Latin America herein analyzed significantly impacts HRQoL and psychological disorders. These findings are consistent with those from Sperber et al. [8], who observed that the overlap of painful and non‐painful disorders had a greater impact on quality of life and psychometric scores compared to the diagnosis of a single DGBI category in individuals.

Our study has some limitations. While the division of all DGBI into painful and non‐painful categories might be viewed as an excessive simplification of the broad range of DGBI diagnoses and, therefore, a limitation, this approach is not unprecedented [11] and is supported by both pathophysiological and epidemiological evidence [14]. Moreover, although the observed differences in mean scores for HRQoL, anxiety, and depression were consistent with the conceptual hypothesis that painful DGBI negatively impact these outcomes, assessing the clinical relevance of these differences is challenging. The cross‐sectional nature of the study also introduces risks of recall bias and a lack of symptoms follow‐up. Additionally, the study did not have access to participants' medical records, so the exclusion of individuals with “organic” gastrointestinal symptoms was based on a checklist completed by the participants. Another study limitation is that the average years of schooling in the studied population were higher than the average observed in the general population of the evaluated countries (14.5 vs. 9.7–11.2 for males and 9.7–11.4 for females in 2022 across the four countries) [34]. This discrepancy may make it difficult to generalize the findings to the broader population of these countries. Nevertheless, our study also has several strengths, including rigorous and standardized data collection methodology, the use of validated questionnaires for the analysis of quality of life and psychological disorders and the inclusion of a multinational sample.

## 5 Conclusion

In conclusion, our study demonstrated that individuals with painful DGBI seek medical care more frequently and exhibit worse quality of life and higher psychological symptom scores compared to those with non‐painful disorders. Female sex and living in communities with fewer than 50,000 inhabitants were independent factors associated with worse physical and mental quality of life scores, with female sex having the greatest impact on somatization. These findings suggest that people with painful DGBI require special attention, particularly considering recent evidence indicating that pain may be associated with the beginning and persistence of other DGBI symptoms [14].

## Author Contributions

Rômulo Marx: designed the research study, performed the research, analyzed the data, paper writing. Antonio Barros Lopes: designed the research study, performed the research, analyzed the data, and wrote the paper. Rafael da Veiga Chaves Picon: designed research study, analyzed data, paper writing. Suzi Alves Camey: analyzed data, paper writing. Olafur Palsson: database curation, data analysis, original study conceptualization, data analysis. Shrikant I. Bangdiwala: database curation, original study conceptualization, analyzed data. Ami D. Sperber: database curation, original study conceptualization, analyzing data, paper writing. Albis Hani: database curation, original study conceptualization, analyzed data. Luis Bustos Fernandez: database curation, original study conceptualization, analyzed data. Max Schmulson: database curation, original study conceptualization, paper writing. Carlos Francisconi: original study conceptualization, design of research study, performance of research, data analysis, paper writing.

## Conflicts of Interest

The authors declare no conflicts of interest.

## Supporting information


**Table S1:** Classification of DGBI as painful and non‐painful disorders of gut‐brain interaction.
**Table S2:** Distribution of Frequency of Doctor Visits (S14) by Painful and non‐painful DGBI in Four Latin American Countries (Argentina, Brazil, Colombia, and Mexico; *n* = 2852).

## Data Availability

The data that support the findings of this study are available from the corresponding author upon reasonable request.

## References

[1] A. D. Sperber , S. I. Bangdiwala , D. A. Drossman , et al., “Worldwide Prevalence and Burden of Functional Gastrointestinal Disorders, Results of Rome Foundation Global Study,” Gastroenterology 160, no. 1 (2021): 99–114, 10.1053/j.gastro.2020.04.014. 32294476

[2] N. J. Talley , “Functional Gastrointestinal Disorders as a Public Health Problem,” Neurogastroenterology and Motility 20, no. Suppl 1 (2008): 121–129, 10.1111/j.1365-2982.2008.01097.x. 18402649

[3] D. A. Drossman , “Functional Gastrointestinal Disorders: History, Pathophysiology, Clinical Features and Rome IV,” Gastroenterology 2 (2016): 32, 10.1053/j.gastro.2016.02.032. 27144617

[4] E. M. R. Hillestad , A. van der Meeren , B. H. Nagaraja , et al., “Gut Bless You: The Microbiota‐Gut‐Brain Axis in Irritable Bowel Syndrome,” World Journal of Gastroenterology 28, no. 4 (2022): 412–431, 10.3748/wjg.v28.i4.412. 35125827 PMC8790555

[5] T. Vanuytsel , P. Bercik , and G. Boeckxstaens , “Understanding Neuroimmune Interactions in Disorders of Gut‐Brain Interaction: From Functional to Immune‐Mediated Disorders,” Gut 72, no. 4 (2023): 787–798, 10.1136/gutjnl-2020-320633. 36657961 PMC10086308

[6] M. Kano , P. Dupont , Q. Aziz , and S. Fukudo , “Understanding Neurogastroenterology From Neuroimaging Perspective: A Comprehensive Review of Functional and Structural Brain Imaging in Functional Gastrointestinal Disorders,” Journal of Neurogastroenterology and Motility 24, no. 4 (2018): 512–527, 10.5056/jnm18072. 30041284 PMC6175554

[7] L. Van Oudenhove , “Understanding Gut‐Brain Interactions in Gastrointestinal Pain by Neuroimaging: Lessons From Somatic Pain Studies,” Neurogastroenterology and Motility 23, no. 4 (2011): 292–302, 10.1111/j.1365-2982.2010.01666.x. 21255193

[8] A. D. Sperber , T. Freud , I. Aziz , et al., “Greater Overlap of Rome IV Disorders of Gut‐Brain Interactions Leads to Increased Disease Severity and Poorer Quality of Life,” Clinical Gastroenterology and Hepatology 20, no. 5 (2022): e945–e956, 10.1016/j.cgh.2021.05.042. 34052391

[9] E. N. Madva , K. Staller , J. C. Huffman , et al., “Psychiatric Comorbidities Among Adult Patients With Disorders of Gut‐Brain Interaction: Prevalence and Relationships to Treatment Outcomes,” Neurogastroenterology and Motility 35, no. 2 (2023): e14493, 10.1111/nmo.14493. 36371707 PMC9892339

[10] S. R. Knowles , D. Skvarc , A. C. Ford , et al., “Negative Impact of Disorders of Gut‐Brain Interaction on Health‐Related Quality of Life: Results From the Rome Foundation Global Epidemiology Survey,” Gastroenterology 164, no. 4 (2023): 655–668, 10.1053/j.gastro.2022.12.009. 36565940

[11] Å. Frändemark , H. Törnblom , J. P. Hreinsson , et al., “Work Productivity and Activity Impairment in Disorders of Gut‐Brain Interaction: Data From the Rome Foundation Global Epidemiology Study,” United European Gastroenterology Journal 11, no. 6 (2023): 503–513, 10.1002/ueg2.12425. 37332146 PMC10337740

[12] V. Yu , S. Ballou , R. Hassan , et al., “Abdominal Pain and Depression, Not Bowel Habits, Predict Health Care Utilization in Patients With Functional Bowel Disorders,” American Journal of Gastroenterology 116, no. 8 (2021): 1720–1726, 10.14309/ajg.0000000000001306. 34003175

[13] J. Sjölund , I. Kull , A. Bergström , et al., “Quality of Life and Bidirectional Gut‐Brain Interactions in Irritable Bowel Syndrome From Adolescence to Adulthood,” Clinical Gastroenterology and Hepatology 22, no. 4 (2024): 858–866, 10.1016/j.cgh.2023.09.022. 37802270

[14] H. Burton‐Murray , L. Guadagnoli , I. A. Vanzhula , et al., “Pain Is a Cardinal Symptom Cutting Across Rome IV Anatomical Categories in Disorders of Gut‐Brain Interaction: A Network‐Based Approach,” Neurogastroenterology and Motility 36, no. 10 (2024): e14877, 10.1111/nmo.14877. 39077969 PMC12212471

[15] Y. Luo , S. A. Camey , S. I. Bangdiwala , O. S. Palsson , A. D. Sperber , and L. A. Keefer , “Global Patterns of Prescription Pain Medication Usage in Disorders of Gut‐Brain Interactions,” Neurogastroenterology and Motility 35, no. 1 (2023): e14457, 10.1111/nmo.14457. 36111642 PMC10078418

[16] M. J. Schmulson , G. A. Puentes‐Leal , L. Bustos‐Fernández , et al., “Comparison of the Epidemiology of Disorders of Gut‐Brain Interaction in Four Latin American Countries: Results of the Rome Foundation Global Epidemiology Study,” Neurogastroenterology and Motility 35, no. 6 (2023): e14569, 10.1111/nmo.14569. 36989176

[17] “PROMIS Global‐10,” accessed December 28, 2024, https://www.codetechnology.com/promis‐global‐10/.

[18] P. Apputhurai , O. S. Palsson , S. I. Bangdiwala , A. D. Sperber , A. Mikocka‐Walus , and S. R. Knowles , “Confirmatory Validation of the Patient Health Questionnaire–4 (PHQ‐4) for Gastrointestinal Disorders: A Large‐Scale Cross‐Sectional Survey,” Journal of Psychosomatic Research 180 (2024): 111654, 10.1016/j.jpsychores.2024.111654. 38569449

[19] R. C. Spiller , D. J. Humes , E. Campbell , et al., “The Patient Health Questionnaire 12 Somatic Symptom Scale as a Predictor of Symptom Severity and Consulting Behaviour in Patients With Irritable Bowel Syndrome and Symptomatic Diverticular Disease,” Alimentary Pharmacology & Therapeutics 32, no. 6 (2010): 811–820, 10.1111/j.1365-2036.2010.04402.x. 20629976

[20] N. J. Talley , P. M. Boyce , and M. Jones , “Predictors of Health Care Seeking for Irritable Bowel Syndrome: A Population Based Study,” Gut 41, no. 3 (1997): 394–398, 10.1136/gut.41.3.394. 9378398 PMC1891476

[21] N. A. Koloski , N. J. Talley , S. S. Huskic , and P. M. Boyce , “Predictors of Conventional and Alternative Health Care Seeking for Irritable Bowel Syndrome and Functional Dyspepsia,” Alimentary Pharmacology & Therapeutics 17, no. 6 (2003): 841–851, 10.1046/j.1365-2036.2003.01498.x. 12641507

[22] W. J. Fan , D. Xu , M. Chang , et al., “Predictors of Healthcare‐Seeking Behavior Among Chinese Patients With Irritable Bowel Syndrome,” World Journal of Gastroenterology 23, no. 42 (2017): 7635–7643, 10.3748/wjg.v23.i42.7635. 29204063 PMC5698256

[23] T. Fairlie , A. Shah , N. J. Talley , et al., “Overlap of Disorders of Gut‐Brain Interaction: A Systematic Review and Meta‐Analysis,” Lancet Gastroenterology & Hepatology 8, no. 7 (2023): 646–659, 10.1016/S2468-1253(23)00102-4. 37211024

[24] K. Kawai , A. T. Kawai , P. Wollan , and B. P. Yawn , “Adverse Impacts of Chronic Pain on Health‐Related Quality of Life, Work Productivity, Depression and Anxiety in a Community‐Based Study,” Family Practice 34, no. 6 (2017): 656–661, 10.1093/fampra/cmx034. 28444208 PMC6260800

[25] S. A. Walter , M. P. Jones , N. J. Talley , et al., “Abdominal Pain Is Associated With Anxiety and Depression Scores in a Sample of the General Adult Population With no Signs of Organic Gastrointestinal Disease,” Neurogastroenterology and Motility 25, no. 9 (2013): 541–e576, 10.1111/nmo.12155. 23692044

[26] K. K. Garnæs , S. Mørkved , T. Tønne , L. Furan , O. Vasseljen , and H. H. Johannessen , “Mental Health Among Patients With Chronic Musculoskeletal Pain and Its Relation to Number of Pain Sites and Pain Intensity, a Cross‐Sectional Study Among Primary Health Care Patients,” BMC Musculoskeletal Disorders 23, no. 1 (2022): 1115, 10.1186/s12891-022-06051-9. 36544130 PMC9773452

[27] S. D. Mathias , M. Kuppermann , R. F. Liberman , R. C. Lipschutz , and J. F. Steege , “Chronic Pelvic Pain: Prevalence, Health‐Related Quality of Life, and Economic Correlates,” Obstetrics and Gynecology 87, no. 3 (1996): 321–327, 10.1016/0029-7844(95)00458-0. 8598948

[28] J. K. Zia , A. Lenhart , P. L. Yang , et al., “Risk Factors for Abdominal Pain‐Related Disorders of Gut‐Brain Interaction in Adults and Children: A Systematic Review,” Gastroenterology 163, no. 4 (2022): 995–1023, 10.1053/j.gastro.2022.06.028. 35716771 PMC9509486

[29] R. Choghakhori , A. Abbasnezhad , R. Amani , and M. Alipour , “Sex‐Related Differences in Clinical Symptoms, Quality of Life, and Biochemical Factors in Irritable Bowel Syndrome,” Digestive Diseases and Sciences 62, no. 6 (2017): 1550–1560, 10.1007/s10620-017-4554-6. 28374085

[30] A. Mulak , T. Freud , M. Waluga , S. I. Bangdiwala , O. S. Palsson , and A. D. Sperber , “Sex‐ and Gender‐Related Differences in the Prevalence and Burden of Disorders of Gut‐Brain Interaction in Poland,” Neurogastroenterology and Motility 35, no. 6 (2023): e14568, 10.1111/nmo.14568. 36989186

[31] Y. Jiang , B. Greenwood‐Van Meerveld , A. C. Johnson , and R. A. Travagli , “Role of Estrogen and Stress on the Brain‐Gut Axis,” American Journal of Physiology. Gastrointestinal and Liver Physiology 317, no. 2 (2019): G203–G209, 10.1152/ajpgi.00144.2019. 31241977 PMC6734369

[32] A. Maguire and D. O'Reilly , “Does Conurbation Affect the Risk of Poor Mental Health? A Population Based Record Linkage Study,” Health & Place 34 (2015): 126–134, 10.1016/j.healthplace.2015.05.003. 26022773

[33] A. J. Stier , K. E. Schertz , N. W. Rim , et al., “Evidence and Theory for Lower Rates of Depression in Larger US Urban Areas,” Proceedings of the National Academy of Sciences of the United States of America 118, no. 31 (2021): e2022472118, 10.1073/pnas.2022472118. 34315817 PMC8346882

[34] Oficina para América Latina y el Caribe del IIPE UNESCO , “Sistema de Información de Tendencias Educativas en América Latina (SITEAL),” accessed December 28, 2024, https://siteal.iiep.unesco.org/.

