# Supplementary material for: Painful Disorders of Gut‐Brain Interaction Are More Associated With Worse Health‐Related Quality of Life and Psychological Disorders Than Non‐Painful Disorders in Latin American Countries
Source: Neurogastroenterol Motil. 2025 Nov 10;37(12):e70194. doi: 10.1111/nmo.70194 (PMC12623289; doi:10.1111/nmo.70194)
Supplement: Supplementary file 1 — Table S1: Classification of DGBI as painful and non‐painful disorders of gut‐brain interaction. Table S2: Distribution of Frequency of Doctor Visits (S14) by Painful and non‐painful DGBI in Four Latin American Countries (Argentina, Brazil, Colombia, and Mexico; n = 2852). [file NMO-37-e70194-s001.docx]

1. **SUPPLEMENTARY MATERIAL**

TABLE S1: Classification of DGBI as painful and nonpainful disorders of gut-brain interaction

| Painful DGBI | Nonpainful DGBI |
| --- | --- |
| Functional chest pain | Globus |
| Functional heartburn | Functional dysphagia |
| Reflux hypersensitivity | Belching |
| Functional dyspepsia: Epigastric pain syndrome | Functional Abdominal Bloating |
| Irritable bowel syndrome | Rumination |
| Centrally mediated abdominal pain syndrome | Chronic nausea and vomiting |
| Functional biliary pain | Cyclic vomiting |
| Proctalgia fugax | Cannabinoid hyperemesis |
| Levator ani syndrome | Postprandial distress syndrome |
|  | Functional diarrhea |
|  | Functional constipation |
|  | Opioid-induced constipation |
|  | Fecal incontinence |

TABLE S2: Distribution of Frequency of Doctor Visits (S14) by Painful and nonpainful DGBI in Four Latin American Countries (Argentina, Brazil, Colombia, and Mexico; n=2852)

| How often do you go to a doctor for your health? | | | | | |
| --- | --- | --- | --- | --- | --- |
| Once a month or more* | | | | | |
| Painful DGBI | | | Nonpainful DGBI | | |
| n | cases | % (95% CI) | n | cases | % (95% CI) |
| 1132 | 210 | 18.55 (16.33- 20.94) | 1720 | 257 | 14.94 (13.29-16.72) |

* p value 0.0125
